# The Cryoprotective Effect of an Antifreeze Collagen Peptide Complex Obtained by Enzymatic Glycosylation on Tilapia

**DOI:** 10.3390/foods13091319

**Published:** 2024-04-25

**Authors:** Shouchun Liu, Luyao Zhang, Zhuyi Li, Jing Chen, Yinyu Zhang, Xuebo Yang, Qiuhan Chen, Hongying Cai, Pengzhi Hong, Chunhua Zhu, Saiyi Zhong

**Affiliations:** 1 College of Food Science and Technology, Guangdong Ocean University, Guangdong Provincial Key Laboratory of Aquatic Product Processing and Safety, Guangdong Province Engineering Laboratory for Marine Biological Products, Guangdong Provincial Engineering Technology Research Center of Seafood, Guangdong Provincial Engineering Technology Research Center of Prefabricated Seafood Processing and Quality Control, Zhanjiang 524088, China; liusc_zjwlab@163.com (S.L.); s2416001752@163.com (Z.L.); 15292604455@163.com (J.C.); yu13265993076@126.com (Y.Z.); 18193642272@163.com (X.Y.); chenqiuh1120@126.com (Q.C.); hongpengzhi@126.com (P.H.); 2 Southern Marine Science and Engineering Guangdong Laboratory (Zhanjiang), Zhanjiang 524004, China; 15646705235@163.com (L.Z.); 13414866246@163.com (H.C.); zhu860025@163.com (C.Z.); 3 College of Fisheries, Guangdong Ocean University, Zhanjiang 524088, China

**Keywords:** tilapia, fish collagen peptide, transglutaminase, glycosylation, antifreeze

## Abstract

Antifreeze peptides have become effective antifreeze agents for frozen products, but their low quantity of active ingredients and high cost limit large-scale application. This study used the glycosylation of fish collagen peptides with glucosamine hydrochloride catalyzed by transglutaminase to obtain a transglutaminase-catalyzed glycosylation product (TGP) and investigate its antifreeze effect on tilapia. Compared with the blank group, the freshness (pH value of 6.31, TVB-N value of 21.7 mg/100 g, whiteness of 46.28), textural properties (especially hardness and elasticity), and rheological properties of the TGP groups were significantly improved. In addition, the protein structures of the samples were investigated using UV absorption and fluorescence spectroscopy. The results showed that the tertiary structure of the TGP groups changed to form a dense polymer. Therefore, this approach can reduce the denaturation and decomposition of muscle fibers and proteins in fish meat more effectively and has a better protective effect on muscle structure and protein aggregation, improving the stability of fish meat. This study reveals an innovative method for generating antifreeze peptides by enzymatic glycosylation, and glycosylated fish collagen peptide products can be used as new and effective green antifreeze agents in frozen foods.

## 1. Introduction

Tilapia fish meat is rich in protein, trace elements, vitamins, and has a unique flavor; thus, many consumers love tilapia [1]. With the continuous development of tilapia products, preservation methods for these fish deserve attention. Since tilapia muscle contains high moisture, protein, and polyunsaturated fatty acids levels, its quality decreases rapidly during storage [1]. Therefore, studying changes in freshness during storage is essential for maintaining food quality. Food freezing is a common storage method for processed fish. Freezing at temperatures between −18 and −30 °C slows biological and chemical reactions and reduces the physical degradation of fish, including enzyme activity, color changes, and lipid oxidation [2]. However, deterioration of fish quality, such as changes in texture, color, and flavor, is still unavoidable under freezing conditions. In particular, freeze–thaw (F-T) cycles caused by inevitable temperature fluctuations during low-temperature transportation and circulation, storage, and consumption exacerbate microbial spoilage and protein changes [3], thus affecting the quality of tilapia.

Food quality loss during frozen storage is caused mainly by mechanical damage from ice crystal growth [4]. To address the quality loss caused by the freezing process, cryoprotectants have been investigated and applied to meat to improve water retention and resistance to protein denaturation. Antifreeze agents such as antifreeze peptides, sugars, and complex phosphates have been shown to bind to ice and inhibit its growth [5]; of these, antifreeze peptides serve as a green and effective cryoprotectant for food without causing unwanted sweetness [6] or, moreover, harming human health [7]. For example, Cao et al. [6] reported that tilapia-skin collagen hydrolysate exhibited good antifreeze activity, and its antifreeze effect was comparable to commercially available phosphorus antifreeze. Collagen hydrolysates of shark-skin gelatin can prevent protein denaturation by reducing protein aggregation during freezing and thawing, thus contributing to fish cryoprotection [8]. Damodaran et al. [9] found that short collagen/gelatin peptides with molecular masses between 1000 and 2500 Da effectively inhibited ice crystal growth in ice cream. Although antifreeze peptides can greatly satisfy the requirements for food antifreeze treatment, compared with traditional antifreeze, the high dosage and high cost of antifreeze peptides limit their development in the food industry. If antifreeze peptide and sugar are combined, their synergistic antifreeze effect significantly increases and simultaneously meets food safety and health requirements for antifreeze treatment in fish.

Glycosylation can effectively regulate the interactions between protein molecules and has been widely used to modify the functional properties of proteins. Transglutaminase (TGase)-induced glycosylation is the most commonly used method. Proteins can be covalently bound to sugars by enzymatic glycosylation methods to enhance their functional properties and stability [10]. For instance, Yang et al. [11] prepared oligochitosan-modified ferritin nanoparticles using TGase-induced glycosylation. They found that glycosylated ferritin was superior to untreated ferritin for encapsulating and stabilizing bioactive molecules in food and nutrition. Zhang et al. [12] prepared oligochitosan-modified alginate–red protein complexes by binding TGase-catalyzed oligochitosan to the alginate–red protein complex. The complexes had good stability and improved the application of algal green protein as a natural colorant in food. Wang et al. [13] prepared hemolysin glycopeptides from TGase-induced D-glucosamine and found that glycosylated hemolysin peptides contained more amide bonds and hydrophobic amino acids, which could protect against acute alcoholic liver injury. Zhang et al. [14] used TGase to incorporate glucosamine into soybean isolate proteins. They found that glycosylated soybean isolate proteins improved the F-T stability of emulsions and effectively ameliorated oil droplet aggregation. These protein glycosylation products combine the macromolecular properties of proteins and the hydrophilic properties of sugars, resulting in good solubility, emulsification, gelation, and thermal stability. To the best of our knowledge, the effect of TGase-induced glycosylation on the antifreeze properties of collagen peptides has been less explored. In contrast, improving the quality characteristics of frozen aquatic products has always been a priority, and developing novel, effective, and safe antifreeze agents is a potentially valuable endeavor.

Therefore, in this study, a transglutaminase-catalyzed glycosylation product (TGP) was prepared from fish collagen peptides and glucosamine hydrochloride, and its antifreezing effect on the frozen storage of tilapia was investigated. The study examined the impact of the TGP on the protein structure and quality of tilapia fillets, as well as the antifreeze effect of glycosylation on F-T tilapia. This work aimed to explain whether TGPs can improve the quality of frozen fish fillets and to expand the application of the protease glycosylation method as an antifreeze technology in the frozen food industry.

## 2. Materials and Methods

### 2.1. Materials

Fresh tilapia (average length: 30 ± 5 cm, weight: 1300 ± 20 g) was purchased from Dongfeng Market (Zhanjiang, China). Fish collagen peptide was purchased from Ruikanglai Technology Co., Ltd. (Nanjing, China); glucosamine hydrochloride was purchased from Cohen Biotechnology Co., Ltd. (Yangzhou, China); transglutaminase and bovine serum albumin were purchased from Yuanye Biotechnology Co., Ltd. (Shanghai, China); sodium chloride, calcium chloride, and ethanol were purchased from Guanghua Technology Co., Ltd. (Guangdong, China); and SDS–PAGE electrophoresis solution was purchased from Biyuntian Biotechnology Co., Ltd. (Shanghai, China).

### 2.2. Sample Preparation

The impregnation liquid was prepared following Hong et al. [15], with slight modifications. Approximately 1.0 g of fish collagen peptide, 1.0 g of glucosamine hydrochloride, and 2 units/g of fish collagen peptide from TGase activation solution (5 mM calcium chloride solution activation) were dissolved in 20 mL of distilled water. Then, TGP was obtained by heating in a 40 °C water bath for 3.5 h. The amount of TGase added was adjusted to prepare experimental groups with 1%, 3%, and 5% impregnating solution. Simultaneously, the unsoaked samples were used as the blank group, while the control group (CG) consisted of 3% fish collagen peptide and glucosamine hydrochloride.

Sample treatment: Tilapia fish were picked and cut into uniformly sized fillets (10 cm × 6 cm × 1.5 cm), and roughly 200 g of fillets were placed in a vacuum-impregnated vessel and soaked for 30 min (three groups of fish were impregnated with each antifreeze). The fillet samples were frozen at −18 °C for 48 h and then thawed at 4 °C for 12 h. Next, the samples were frozen at −18 °C for 24 h and thawed at 4 °C for 12 h for subsequent experimental tests. Three fillets were taken from each dipping group for the determination of indicators, and each indicator was determined at least three times.

### 2.3. Determination of Freshness

#### 2.3.1. pH

At total of 10.0 g of tilapia fillets was accurately weighed after the F-T cycle and mixed with 100 mL of distilled water, with magnetic stirring for 30 min to ensure homogeneity of the mixture. The pH of the solution was determined using a pH meter (PHA-3C, INESA Scientific Instruments Co., Ltd., Shanghai, China). Each experiment was repeated three times for accuracy.

#### 2.3.2. Total Volatile Basic Nitrogen (TVB-N)

The determination of volatile basic nitrogen was carried out according to the automatic Kjeldahl nitrogen analyzer method of GB 5009.228-2016 [16]. Each tilapia fillet (10.0 g) subjected to F-T cycles was combined with 75 mL distilled water, placed in a distillation tube, shaken to mix evenly, and impregnated for 30 min for later use. After shaking, 1.0 g of magnesium oxide was added to the distillation tube and then immediately measured. The distilled nitrogen-containing substances were absorbed with absorption solution. Then, they were immediately titrated with 0.01 mol/L hydrochloric acid standard solution. According to the consumption of standard solution, the content of volatile base nitrogen in the sample was able to be calculated.

The detection conditions of the instrument were as follows: absorbent: 30 mL boric acid solution (2%, *w*/*v*); mode time: 5 s; distillation time: 5 min; and steam amount: 100%.

#### 2.3.3. Color Difference

Fish fillets (1 cm × 1 cm) were placed in the carrier dish of the colorimeter, and the colorimeter was used to measure the change in color of the fish surface. L* is the brightness value of the sample, a* is the red–green value, and b* is the yellow–blue value. The whiteness formula is as follows (1):

Whiteness = 100 − [(100 − L*)^2^ + a*^2^ + b*^2^] ^1/2^ (1)

### 2.4. Moisture Change

#### 2.4.1. Drip Loss

Before impregnation treatment, the tilapia fillets were weighed, and the weight was recorded as m_1_. After F-T cycles, the tilapia fillets were dried with absorbent paper to dry surface moisture, and the weight was recorded as m_2_. The drip loss was calculated according to the following Equation (2):

Drip loss rate (%) = (m_1_ − m_2_)/m_1_ × 100 (2)

#### 2.4.2. Low-Frequency Nuclear Magnetic Resonance (LF-NMR)

Moisture distribution was determined using LF-NMR (NMI20-025V-I, Niumag Electric Corporation, Shanghai, China) according to the methods of Wang et al. [17]. Five tilapia fillets of uniform size and thickness were selected and placed one by one in an NMR tube for determination. The program was set as follows: magnet temperature: 32 °C; sampling points: 6160; τ: 120 µs; scanning number: 6; and echo number: 8000. The data were analyzed using the inversion software MultiExp Inv (Niumag Electric Corporation, Shanghai, China).

### 2.5. Protein Structure

#### 2.5.1. Extraction of Myofibrillar Protein (MP)

MP was prepared according to the method of Peng et al. [18], with slight modifications. A total of 5.0 g surimi was mixed with 50 mL of PBS buffer (pH: 7.0) then homogenized at 8000 r/min for 1 min and centrifuged at 7000× *g* r/min at 4 °C for 15 min, after which the residue was taken and washed twice. The residue was then added to the threefold volume of PBS (0.6 mol/L KCl, pH: 7.0), homogenized for 1 min, and left to stand for 1 h at 4 °C. The supernatant obtained by centrifugation at 8000× *g* r/min for 20 min was the MP solution.

#### 2.5.2. Sodium Dodecyl Sulfate–Polyacrylamide Gel Electrophoresis (SDS–PAGE)

The concentration of the MP solution was adjusted to 1 mg/mL, mixed with SDS-PAGE sample buffer (2×), brought to a boil, and loaded into the prepared gel. The electrophoresis equipment was assembled, and electrophoresis was stopped when the bands reached the bottom of the separator gel. The resulting electropherogram was then observed on a gel imager.

#### 2.5.3. Tertiary Structural Changes in MP

A total of 200 μL of 0.5 mg/mL MP was added to 96-well plates, and the UV absorption spectrum was detected according to the methods of Chen et al. [19], with slight improvements. The detection mode of the microplate reader was UV spectroscopy, and the detection wavelength was in the interval of 200~400 nm. The method of detecting endogenous fluorescence spectroscopy signals was performed according to the methods of Cao et al. [20], with slight improvements. The detection mode was endogenous fluorescence spectroscopy, and the parameters were set as follows: slit width: 10 mm; sensitivity: 3; excitation wavelength: 295 nm; and emission interval: 300~400 nm.

### 2.6. Texture Property Analysis (TPA)

The thawed dorsal muscles were divided into 2 cm × 2 cm × 1 cm fish pieces and placed in a texture analyzer (TMS-PRO, Beijing HuaTaiTong Science & Technology Co., Ltd., Beijing, China) to determine their textural characteristics (hardness, gumminess, springiness, and resilience). The test parameters were as follows: pretest speed: 2 mm/s; mid-test speed: 1 mm/s; posttest speed: 2 mm/s; strain: 50%; interval time between two tests: 2 s; trigger force: 5 g; probe type: P/10 (diameter, 10 mm); and target mode: strain.

### 2.7. Dynamic Rheology

The dynamic rheology was determined according to the methods of Huang et al. [21], with slight modifications. Approximately 1.0 g of sample was placed on the loading table of the rheometer, and a parallel plate of 35 mm was used for determination. The slit spacing was set to 1 mm, and the surface of the sample was sealed with paraffin oil to avoid dehydration during the determination. The temperature scanning parameters were as follows: sample equilibrium time: 20 min; temperature: increased from 25 °C to 80 °C at a rate of 2 °C/min; frequency: 1 Hz; and strain: 0.1%. The frequency scanning parameters were as follows: temperature: 25 °C; strain: 0.1%; and frequency range: 0.1–20 Hz. The strain scanning parameters were as follows: temperature: 25 °C; frequency: 1 Hz; and strain range: 0.01–100%.

### 2.8. Statistical Analysis

Experimental data were analyzed by ANOVA and shown as mean ± standard deviation. Comparative analysis of means was performed using Duncan’s multiple test. Statistical analysis was performed using SPSS 17.0. All experiments were independently replicated at least three times.

## 3. Results and Discussion

### 3.1. Physicochemical Properties

#### 3.1.1. pH

**Figure 1:**
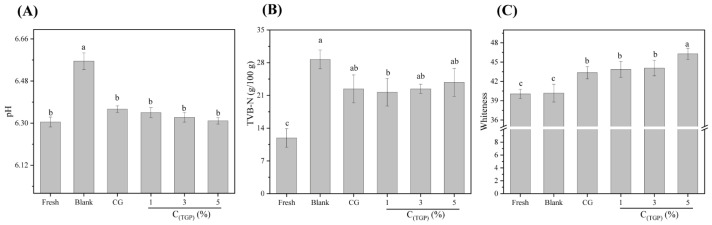
Physicochemical properties of different tilapia samples: (**A**) pH; (**B**) TVB-N; (**C**) whiteness (*n* = 3). Different lowercase letters indicate significant differences between different storage times for the same storage method (*p* < 0.05).

Protein degradation occurs during the storage process, resulting in changes in the pH of the fish so that the pH can represent the freshness of the fish during storage to a certain extent. As shown in Figure 1A, after F-T cycles, the pH of the fish meat in each group increased (6.31 → 6.57). This is due to the temperature changes during F-T cycles that lead to the growth of ice crystals in the food. Recrystallization of ice crystals ruptures the cells, leading to the denaturation of muscle proteins [22], which affects the pH value. Additionally, during storage, the decomposition of proteins and amino acids can produce substances such as ammonia, which further increases the pH value [23]. A higher pH indicates that the spoilage of the sample is relatively severe and the corresponding change in the degree of freshness following the change has occurred. The pH of the samples in the dipping groups was significantly lower than that of samples in the blank group, possibly because the glycosylation products inhibited fish spoilage to a certain extent and had the ability to protect against freezing.

#### 3.1.2. TVB-N

During the storage process of tilapia, alkaline nitrogenous substances such as ammonia and amines are produced in the fish flesh due to the promotion of enzymes and bacterial infections, which in turn affect the quality and freshness of the fish flesh. Therefore, TVB-N has become the most critical indicator of fish freshness. Typically, a TVB-N concentration ≥30 mg/100 g in marine fish and shrimp is considered spoilage [24]. As shown in Figure 1B, the TVB-N content increased after F-T cycles. The formation of ice crystals during repeated F-T treatments led to the rupture of cells and accelerated the oxidative decomposition of proteins [19]. Simultaneously, many nutrients from the cells provided a propagation medium for microorganisms. In addition, the increased activity of endogenous enzymes and spoilage microorganisms and the presence of microbial degradation products exacerbated the accumulation of TVB-N, leading to a high degree of meat spoilage [25]. The TVB-N content of the samples in the blank group had a critical spoilage value (28.9 mg/100 g), while the TVB-N content of the samples in the dipping group was significantly lower than that of the blank group (*p* < 0.05).

Therefore, treating fish meat with a dipping solution suppressed the increase in TVB-N to a certain extent and reduced the degree of spoilage. However, there was no significant difference in the protective effect of the different dipping solutions on the freshness of the fish meat (*p* > 0.05).

### 3.2. Whiteness

The color of fish meat changes during storage due to the degradation or oxidation of pigments or proteins, dramatically affecting the consumer’s senses; therefore, color is also an indicator of fish spoilage. Figure 1C shows the effects of different antifreeze solutions on the color of the fish meat. The whiteness of the samples decreased to varying degrees after F-T cycles, which may be attributed to the temperature fluctuations accelerating the browning reaction and the oxidation of myoglobin in the fish, resulting in different degrees of browning. Under the same freezing conditions, the whiteness value of the dipping group was greater than that of the blank group; in particular, the whiteness value of the 5% TGP group reached the highest value of 46.28. This difference may be attributed to the fact that TGP inhibited myoglobin oxidation in the tilapia to a certain extent and slowed the browning reaction. In addition, the antifreeze’s composition and color also affected the whiteness of the fish.

### 3.3. Water Status

#### 3.3.1. Drip Loss

**Figure 2:**
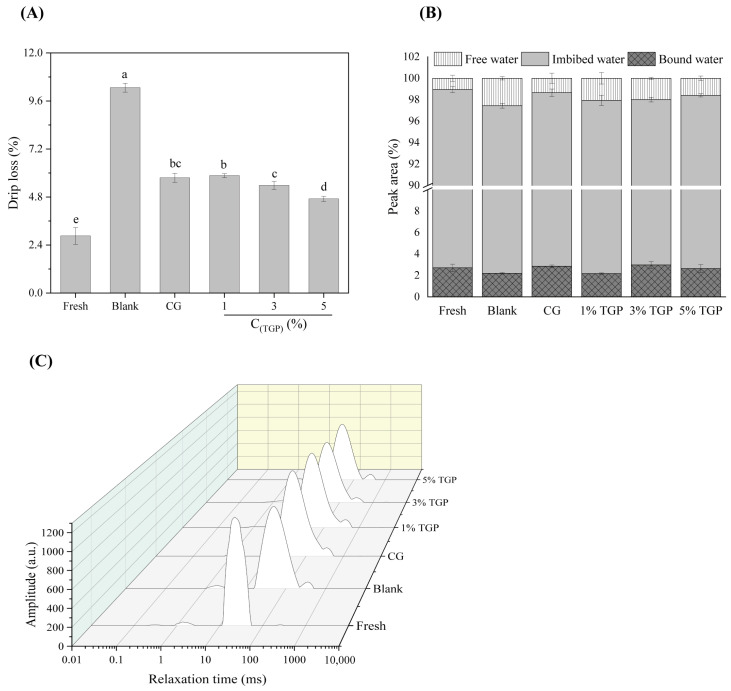
Moisture status of tilapia under different treatments: (**A**) drip loss; (**B**) proportion of peak area in T_2_ relaxation time; (**C**) distribution of relaxation time T_2_ (*n* = 3)_._ Different lowercase letters indicate significant differences between different storage times for the same storage method (*p* < 0.05).

The drip loss of meat products is an essential index for quality evaluation and affects the quality of meat products, such as nutrient content, tenderness, and color. The effect of TGP on drip loss in fish is shown in Figure 2A. The drip loss of fresh fish was 2.86%, and after F-T cycles, the drip loss of fish (10.27%) was much greater than that of fresh fillets (*p* < 0.05). During repeated F-T cycles, different sizes and locations of ice crystals exacerbate cell damage, coupled with protein freeze denaturation and a weakened ability to bind water, resulting in decreased water-holding capacity of the muscle [26]. Both the glycopeptide mixture and TGP reduced the drip loss of the fish, among which the 3% and 5% TGP groups exhibited better ice crystal growth, and the fish lost less water. The decrease in water loss caused by the antifreeze agent was related to the alteration in the MP concentration, which promoted the interaction of proteins with water and thus improved water retention. Sugars are rich in hydroxyl groups that can reduce the destructive effect of ice crystal formation, increase osmotic pressure and other factors affecting the structure of proteins during the freezing process, and have excellent protective effects [27]. Antifreeze peptides lower a solution’s freezing point, altering ice crystal growth and increasing its stability. They inhibit recrystallization by binding to the ice surface and inhibiting ice growth through an adsorption inhibition mechanism [28]. While the TGP contained small-molecule sugars and fish collagen peptides, the -OH and -NH_3_ hydrophilic groups provided pairs of water-binding sites, thus increasing water absorption [29]. Moreover, the TGP inhibited the growth of ice crystals and recrystallization phenomena and increased the viscosity of the solution, therefore slowing the nucleation process and decreasing the formation of ice crystals for antifreeze purposes [19]. The results showed that protein glycosylation products as antifreeze agents could improve water retention.

#### 3.3.2. LF-NMR

The water properties of tilapia fillets can be examined using LF-NMR to characterize their water mobility and distribution. Figure 2B,C show the distributions of the T_2_ relaxation times and the peak area ratios for the different treated samples. Three peaks were detected in all groups of samples: bound water (0.1~10 ms), immobile water (10~100 ms), and free water (100~1000 ms). After F-T cycles, the T_2_ values of the samples all shifted right, indicating increased water molecule mobility [30]. Under repeated F-T cycles, ice crystals of different sizes and locations form, damaging the tissue and MP of the muscle and disrupting the lattice structure of its proteins, which leads to a decrease in the ability of the muscle tissue to retain water [3]. Moreover, a series of physiological activities leads to the destruction of myogenic fibers so that the muscle tissue undergoes water migration. The immobile water was gradually transformed into free water [31]. Compared with those in the blank group, the T_2_ values in the dipped samples migrated to the left, especially in the groups treated with 3% and 5% TGP, indicating that the addition of TGP as an antifreeze agent enhanced the binding strength of water molecules and reduced the degree of water freedom. Moreover, the peak area ratios (Figure 2B) showed a slight increase in the peak area of bound water and a slight decrease in the peak area of free water for the samples in the TGP groups. Taken together, these findings indicate that TGP contains both small-molecule sugars and hydrophilic groups (-OH and -NH_3_, etc.) from fish collagen peptides, which provide additional pairs of water-binding sites, increase the proteins’ ability to hold onto water and reduce the mobility of water molecules, thus improving water retention properties [29].

Combined with the moisture determination results, these findings suggested that adding TGP enhanced the binding of proteins to water molecules in tilapia and reduced water loss. Therefore, TGP could be applied as a novel antifreeze agent for frozen foods.

### 3.4. Protein Structure

#### 3.4.1. SDS–PAGE

**Figure 3:**
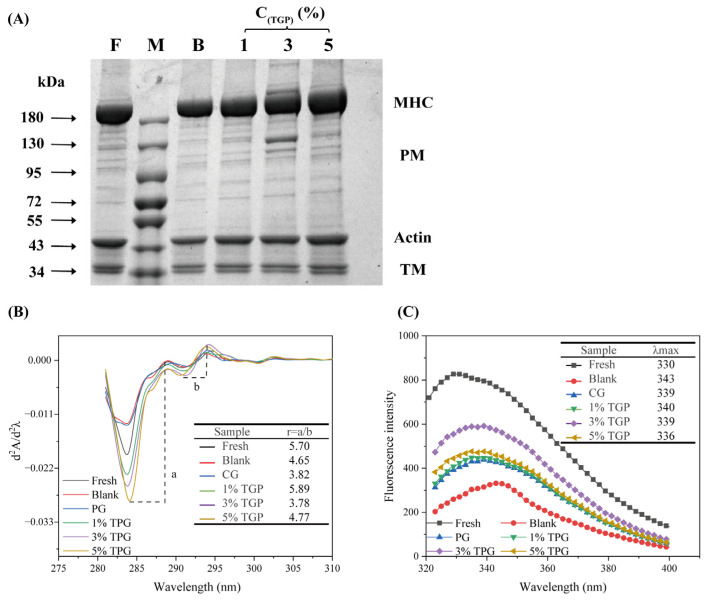
(**A**) SDS-PAGE; F: fresh group, M: marker, B: blank group, MHC: myosin heavy chain, PM: paramyosin, TM: tropomyosin; (**B**) UV absorption spectroscopy (The distances between the valleys and peaks of the two peaks from left to right are a and b, respectively); and (**C**) intrinsic fluorescence spectroscopy of tilapia treated with different methods (*n* = 3).

SDS–PAGE can be used to observe the denaturation, aggregation, and degradation of MPs in F-T tilapia. As shown in Figure 3A, the MPs consisted of a myosin heavy chain (MHC), paramyosin (PM), actin, and tropomyosin, among which myosin was an essential component of the MPs and was mainly responsible for the formation of the protein network [32]. After the F-T cycles, all the MP bands became lighter to varying degrees. The actin and PM bands became smaller and almost disappeared. During repeated F-T cycles, the continuous generation of ice crystals and increase in volume led to the contraction or breakage of myofibrils, which destroyed their integrity and triggered the denaturation of MPs [3], resulting in the discoloration of electrophoretic bands. Compared with those of the blank group, the MHC bands of the dipped samples were deeper and thicker, indicating that the experimental impregnation solution could inhibit the decrease in the concentration of MHC in the fish meat and better protect the fish proteins. Among them, the bands corresponding to the PM and TM in the TGP groups were more apparent, especially in the 3% TGP group. These findings indicate that the use of TGP as a dipping solution could effectively inhibit the degradation of proteins, prevent structural damage, and play a certain role in protein protection.

#### 3.4.2. UV Absorption Spectrum

UV absorption spectroscopy uniquely discriminates aromatic amino acids and is used to determine changes in protein tertiary structure. By calculating the second-order derivatives of the UV absorption spectra, the resolution can be improved to sufficiently reflect changes in amino acid residues and protein structures in the microenvironment [33]. As shown in Figure 3B, there were two positive absorption peaks at 287 nm and 294 nm and two negative absorption peaks at 284 nm and 291 nm. The positions of the peaks of the samples were all redshifted after F-T cycles, indicating that temperature fluctuations denature the proteins, exposing tyrosine and tryptophan residues based on the surface, which increases the hydrophobic interaction force and leads to a shift in the absorption peaks to longer wavelengths [34]. The peak at 287 nm was shifted to a shorter wavelength (blueshift) in the dip-treated samples than in the blank group. In addition, by calculating the ratio between two peaks and valleys (r = a/b), it is possible to determine the relative content of amino acids and the average polarity of proteins in the microenvironment [35]. The decreasing trend in the r value of the samples after freezing and storage indicated that freezing and thawing caused aggregation of the protein structure and decreased the polarity of the microenvironment for tyrosine, but the r value of the samples increased slightly after the soaking treatment, which indicated that the polarity of the environment around the tyrosine and tryptophan residues increased [36]. The r values of the samples in the TGP groups increased to a certain extent, indicating that the TGP changed the microenvironment of tryptophan and tyrosine, stabilized the spatial conformation of the proteins, and effectively reduced the aggregation of proteins in the freeze–thawed fish fillets.

#### 3.4.3. Intrinsic Fluorescence Spectroscopy

Tryptophan has vigorous fluorescence intensity and sensitivity to microenvironmental changes and is often used as an endogenous fluorescent probe to study conformational changes in proteins [37]. The fluorescence intensity of the samples gradually decreased after F-T cycles (Figure 3C), indicating that the amino acids in the side chains of the proteins were oxidized and burst by repeated F-T cycles; meanwhile, the protein structure unfolded, and the internal tryptophan residue groups were exposed on the surface. The intermolecular forces of proteins were enhanced, resulting in aggregation and folding [38], which caused a decrease in the intensity of endogenous fluorescence. This was confirmed by ultraviolet absorption spectroscopy. The most significant reduction in fluorescence peak intensity was observed in the blank group (59.81%), while the peak intensities in the TGP groups were 45.64%, 28.45%, and 43.37%, respectively. In addition, the maximum fluorescence emission wavelength (λmax) of tryptophan reflects the state of the tertiary structure of proteins [25]. The degree of the blueshift of λmax can be used to assess the overall conformational changes in proteins. Repeated F-T treatments altered the λmax redshift of tryptophan in tilapia MP to 343 nm, whereas the redshift was decreased in the dipping group. These phenomena indicated that adding a dipping solution had an antifreeze effect and effectively inhibited the freeze denaturation of proteins, thus stabilizing the tertiary structure of the fish proteins.

### 3.5. TPA

**Figure 4:**
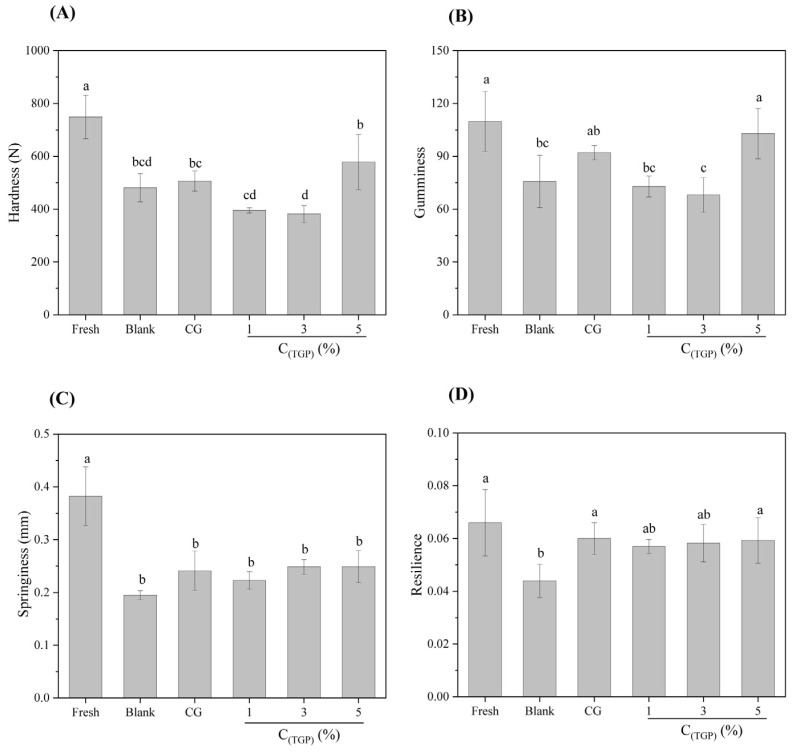
Texture properties: (**A**) hardness; (**B**) gumminess; (**C**) springiness; (**D**) resilience (*n* = 5). Different lowercase letters indicate significant differences between different storage times for the same storage method (*p* < 0.05).

Texture is an essential sensory analysis factor and a critical evaluation index of meat quality. Figure 4 shows the changes in the hardness, gumminess, springiness, and resilience of fish meat during storage. Hardness refers to the force necessary for a sample to achieve a certain deformation. The hardness of the fish meat decreased rapidly after F-T cycles (Figure 4A) due to the formation of ice crystals during F-T cycling, which damaged the cellular tissues and led to the loosening and softening of muscle tissues [39]. The hardness of the samples in the 5% TGP group increased accordingly, but there was no significant difference between the samples in the 5% TGP group and those in the blank group (*p* > 0.05). Combined with the above drip-loss experiments, the changes in the hardness and water-holding capacity of the fish meat exhibited similar trends. In the dipping groups, the water-holding properties were better than those in the blank group, and the hardness decreased more gradually, i.e., the effect of freezing resistance was better. Gumminess is defined as hardness × cohesion and describes the texture of semi-solid foods. The gumminess of the samples improved after dipping, with a significant increase in adhesion in the 5% TGP group (*p* < 0.05) and slight decreases in bonding in the 1% TGP and 3% TGP groups (Figure 4B), which may be due to intersample differences. Springiness is the distance measured when the food regains height between the end of the first bite and the beginning of the second bite. Resilience measures how a sample resumes deformation and includes both speed and force. The groups exhibited no significant differences in the resilience or springiness of the fish (Figure 4C,D).

In summary, the 5% TGP treatment improved the texture and improved the impregnation effect, which was attributed to the more complete glycosylation reaction catalyzed by TGase, and the action of TGP in tilapia flesh could reduce the mechanical damage caused by ice crystal formation to a greater degree, thus improving the textural properties of the fish flesh.

### 3.6. Rheological Properties

#### 3.6.1. Temperature Scanning

**Figure 5:**
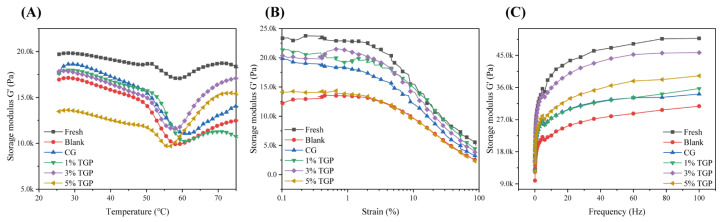
Dynamic rheological properties: (**A**) temperature scanning; (**B**) strain scanning; (**C**) frequency scanning (*n* = 3).

Dynamic rheology can be used to study the solution–gel thermal transition behavior of fish MPs. Figure 5 shows the energy storage modulus (G′) during the heating process of fish meat. The change in G′ was divided into three parts, including a slight increase from 47 to ~52 °C, a sharp decrease when heated to 59 °C, and a rebound when heated to 75 °C. The first peak (approximately 52 °C) was the initial phase of gelation, and the increase in G′ may be due to the denaturation of the myosin head, which is the beginning of the protein–protein interactions that form weak bonds at low temperatures [40]. The sharp decrease in the phase density indicated that thermal denaturation led to the reorganization of proteins and the disruption of the initial network structure [41]. The later elevation stage of G′ meant that there was cross-linking and aggregation of the proteins to form a strong and irreversible gel network structure [42]. After the F-T cycles, the samples showed a similar change in G′ with an overall decrease, indicating that the F-T cycles disrupted the protein structure to different degrees. In contrast, the highest G′ value was found in the 3% TGP group, indicating that the treated fish had better gel properties. As an antifreeze agent, the TGP inhibited the freeze denaturation of proteins to a certain extent, effectively protecting the gel properties of proteins. However, with the addition of TGase, the G′ value decreased, possibly because the high concentration of TGP might affect the rate of the polymerization and denaturation of MPs, which leads to the rough structure of the protein gel network.

#### 3.6.2. Strain Scanning

Dynamic strain experiments can better reveal the structural changes in myofibrillar proteins. As shown in Figure 5B, the samples in each group exhibited the same change trend: destruction and rearrangement of the tight structure between the proteins were observed when the strain increased, and a rapid decrease in the G′ value was observed when the strain was 1%. The dipping groups had higher G′ values than the blank group, and the highest G′ value occurred in the 3% TGP group. This indicated that adding glycosylation products as antifreeze agents created a robust protein network structure in the fillets.

#### 3.6.3. Frequency Scanning

Frequency scans can reflect information about the protein-related network structure. As shown in Figure 5C, the G′ of all the groups increased monotonically throughout the frequency scanning range, which implies that the gelation properties of the three-dimensional gel network of the MPs increased with the increasing scanning frequency. After the F-T cycles, the G′ decreased, which represented a gradual decrease in the strength of the protein gel network. However, the elastic modulus of the impregnated group was greater than that of the blank group, indicating that the impregnation solution could enhance the elasticity of the protein gel network in all the groups. During freezing, the glycopeptide mixture and TGP had specific cryoprotective effects on fish myofibrillar proteins. Among the treatments, 3% TGP had the best cryoprotective effect, followed by the 5% TGP treatment.

To summarize the results of the dynamic rheology experiments, using a 3% TGP impregnation solution could effectively maintain the stability of the spatial structure of the MP. Thus, protein glycosylation was used as a dipping solution for the tilapia dipping treatment, which kept the spatial structure of the fibrillar proteins of the fish muscle fibers and improved the quality of the fish meat.

## 4. Conclusions

This study investigated the cryoprotective effect of fish collagen peptide glycosylation products catalyzed by TGase on tilapia fillets. The results showed that TGP maintained fish freshness and MP integrity during repeated F-T cycles. The freshness, texture, protein properties, and rheological properties of the fish fillets decreased during frozen storage, but the addition of TGP alleviated the deterioration of the fish meat. TGP promoted binding between MP molecules, thereby improving the stability of the resulting conformation, which can be observed from the tertiary structure analysis of the protein. TGP inhibited myofibrillar protein oxidation during frozen storage to a certain extent, inhibiting its functional decline to maintain quality. The enzymatic protein glycosylation product was innovatively introduced into the frozen storage process for fish meat, and its frost resistance was confirmed, which has specific guiding significance for the actual production of frozen aquatic products. However, this technology has yet to be applied to frozen seafood, and its antifreeze effect in actual refrigeration and freezing needs to be further studied at a later stage to maintain the quality of seafood and meet the needs of consumers.

## Data Availability

The original contributions presented in the study are included in the article, further inquiries can be directed to the corresponding author.
